# Spontaneous *shaker* rat mutant – a new model for X-linked tremor/ataxia

**DOI:** 10.1242/dmm.022848

**Published:** 2016-05-01

**Authors:** Karla P. Figueroa, Sharan Paul, Tito Calì, Raffaele Lopreiato, Sukanya Karan, Martina Frizzarin, Darren Ames, Ginevra Zanni, Marisa Brini, Warunee Dansithong, Brett Milash, Daniel R. Scoles, Ernesto Carafoli, Stefan M. Pulst

**Affiliations:** 1 Department of Neurology, University of Utah, Salt Lake City, UT 84112, USA; 2 Department of Biomedical Sciences, University of Padova, Padova, Italy; 3 Bioinformatics Shared Resource, Huntsman Cancer Institute, University of Utah, Salt Lake City, UT 84112, USA; 4 Unit of Molecular Medicine for Neuromuscular and Neurodegenerative Disorders, Department of Neurosciences, Bambino Gesù Children's Hospital, IRCCS, Rome, Italy; 5 Department of Biology, University of Padova, Padova, Italy; 6 Venetian Institute of Molecular Medicine (VIMM), Padova, Italy

**Keywords:** Ataxia, X-linked, *shaker*, PMCA3, *Atp2b3*

## Abstract

The *shaker* rat is an X-linked recessive spontaneous model of progressive Purkinje cell (PC) degeneration exhibiting a shaking ataxia and wide stance. Generation of Wistar Furth (WF)/Brown Norwegian (BN) F1 hybrids and genetic mapping of F2 sib-sib offspring using polymorphic markers narrowed the candidate gene region to 26 Mbp denoted by the last recombinant genetic marker DXRat21 at 133 Mbp to qter (the end of the long arm). In the WF background, the *shaker* mutation has complete penetrance, results in a stereotypic phenotype and there is a narrow window for age of disease onset; by contrast, the F2 hybrid phenotype was more varied, with a later age of onset and likely non-penetrance of the mutation. By deep RNA-sequencing, five variants were found in the candidate region; four were novel without known annotation. One of the variants caused an arginine (R) to cysteine (C) change at codon 35 of the ATPase, Ca^2+^ transporting, plasma membrane 3 (*Atp2b3*) gene encoding PMCA3 that has high expression in the cerebellum. The variant was well supported by hundreds of overlapping reads, and was found in 100% of all affected replicas and 0% of the wild-type (WT) replicas. The mutation segregated with disease in all affected animals and the amino acid change was found in an evolutionarily conserved region of PMCA3. Despite strong genetic evidence for pathogenicity, *in vitro* analyses of PMCA3^R35C^ function did not show any differences to WT PMCA3. Because *Atp2b3* mutation leads to congenital ataxia in humans, the identified *Atp2b3* missense change in the *shaker* rat presents a good candidate for the *shaker* rat phenotype based on genetic criteria, but cannot yet be considered a definite pathogenic variant owing to lack of functional changes.

## Abstract (precis)

**Summary:** The *shaker* rat mutant: a new model for essential tremors and ataxia characterized by Purkinje cell degeneration.

## INTRODUCTION

Hereditary ataxias in humans comprise a diverse group of diseases that affect the cerebellum and various other regions of the nervous system. The classification of these disorders is complex owing to heterogeneity in clinical signs, age of onset, disease progression and mode of inheritance. Here, we describe the genetic analysis of the *shaker* rat, a model of Purkinje cell (PC) degeneration. This mutant arose spontaneously and was observed in Sprague Dawley (SD) outbred stock in 1991 at Saint Louis University, first described by La Regina et al. (1992), and the phenotype of whole-body tremor, ataxia and wide stance designated as ‘*shaker*’. The *shaker* trait was reported as an X-linked recessive trait.

Various animal models of spontaneously occurring mutants that parallel some aspects of human hereditary ataxia have been reported; for example, *weaver*, *lurcher*, *stumbler*, *tottering* and *teetering* mice (Chou et al., 1991; Frankel et al., 1994; Green and Sidman, 1962; Guyer et al., 2002; Meier, 1967; Soha and Herrup, 1995). Although the identification of candidate genes can arise from several methodologies, including whole-transcriptome shotgun sequencing (WTSS, RNA-seq), whole-genome sequencing (WGS) and whole-exome sequencing (WES), knowledge of genetic variation between strains is imperative to the mapping of candidate genes. For rat laboratory strains, information on genetic variation is currently limited to microsatellite markers and a small number of single-nucleotide polymorphisms (SNPs) (Canzian, 1997; Thomas et al., 2003).

Our objectives were to fine-map the *shaker* locus, to identify candidate genes responsible for the *shaker* phenotype and to establish a model system for the investigation of ataxia. Here, we report the finding of a candidate gene, *Atp2b3*, which encodes PMCA3, a plasma membrane Ca^2+^ ATPase transporter that is abundant in human cerebellum. Ca^2+^ signaling regulates some of the most important neuronal functions; thus, the concentration of Ca^2+^ within neurons must be precisely regulated. The plasma membrane Ca^2+^-ATPases (PMCAs or calcium pumps) are essential for this regulation, with two of the four basic PMCA pump isoforms, PMCA2 and PMCA3, being highly expressed in the brain and the cerebellum (Brini and Carafoli, 2009; Eakin et al., 1995; Filoteo et al., 1997; Jensen et al., 2007).

## RESULTS

### Behavior

The *shaker* rat exhibits a wide-based hind-limb stance, ataxic movements and whole-body tremor with onset as early as 9 weeks of age in the Wistar Furth (WF) background. Early in the disease course, a high-frequency tremor was felt upon the handling of the animal but not visible by cage behavior. This high-frequency tremor is best described as a whole-body vibration, similar to that exhibited by an animal in fear. This high-frequency tremor was more difficult to discern once the disease had fully developed and animals were visibly shaking. The generalized tremor involves the entire trunk and body (Movie 1).

Although 90% of affected animals exhibited a marked shaking tremor phenotype by 12 weeks of age, we did not observe any feeding or grooming abnormalities. The *shaker* rat appeared to be more docile than wild-type (WT) rats, exhibited more exploratory behavior and did not have ledge fear. We did not encounter infertility, or gestational or litter-size complications, and lifespan was not impacted.

### Morphology

**Fig. 1.**
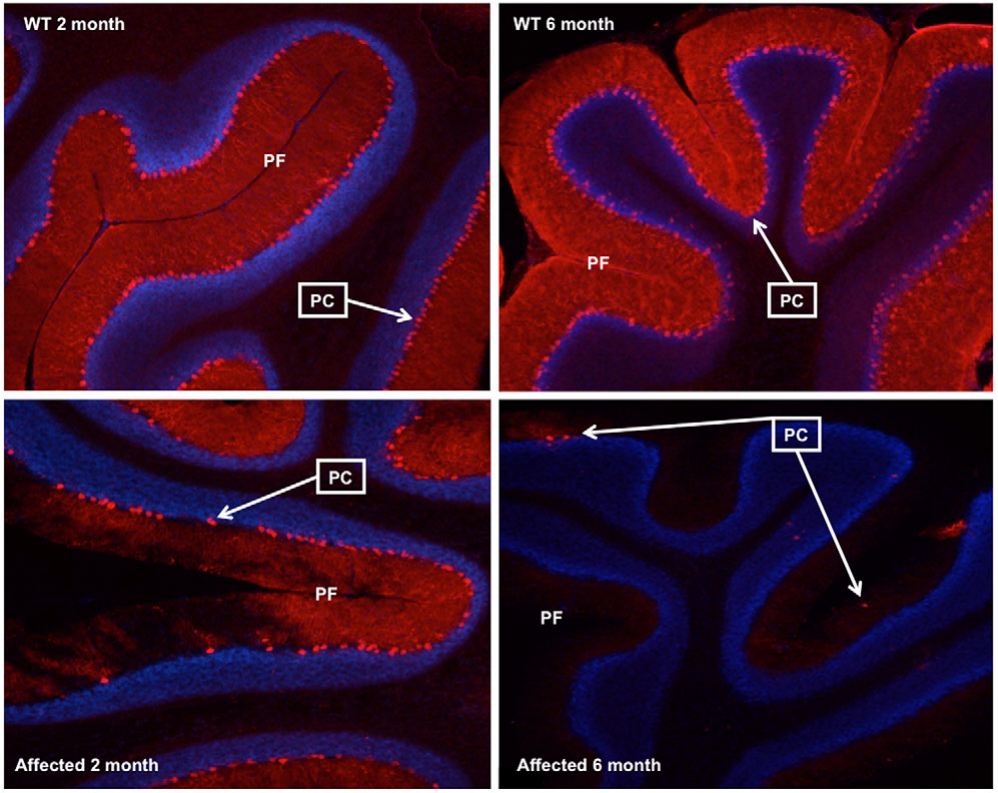
**Significant cerebellar pathology in *shaker* rats.** Three (40 μm) midline cerebellar sections of each group – wild-type (WT) and *shaker* F1 (50/50 WF/BN) hybrid rats – were analyzed at 2 and 6 months of age (*n*=3/group). Sections were stained with an antibody to mouse monoclonal anti-calbindin-D-28K (Calb1; 1:500) and counterstained with DAPI. *Calb1* staining in Purkinje cells (PCs) was reduced in *shaker* 2-month-old animals, and was virtually absent in somata and dendrites in PCs of *shaker* 6-month-old animals. PCs are denoted by arrows. PF, primary fissure.

Significant PC loss in *shaker* cerebella was reported previously in detail (La Regina et al., 1992). We therefore did not conduct a repeat morphometric study. Illustrative samples of midline cerebellar sections at 2 and 6 months of age are shown in Fig. 1. Progressive loss of calbindin staining and loss of PC bodies was evident.

### Genetic mapping

By observation of segregation of the phenotype, X-linked recessive transmission was confirmed. In order to estimate the rate of transmission, we crossed WT males and obligatory carrier females for both WF and F2s. An obligatory carrier female was defined as the female offspring of an affected male crossed with a WT female. We expected a 50/50 ratio of affected and WT males, assuming 100% penetrance by 36 weeks of age. We observed 43/105 (41%) affected males in the WF background and 32/92 (35%) affected males in the F2 cross. Lack of male-to-male transmission on either background further verified X-linked transmission.

Although segregation analysis was consistent with X-linked segregation, we wanted to confirm X-linked inheritance using DNA markers and refine the *shaker* locus using an F2 hybrid mapping strategy. Several recombination events were seen for markers DXRat17 and only one for DXRat21, located at 112,204,377 and 132,940,994, respectively (Table S1). This hybrid mapping strategy mapped the *shaker* locus to a 26-Mbp region of distal rat chromosome X (rat genome version RGSC3.4).

### *Fmr1* repeat expansion

Because *Fmr1* mutation causes a tremor/ataxia phenotype and the *Fmr1* gene is located in the candidate region, we excluded the *Fmr1* repeat expansion as a causative mutation for the *shaker* rat by amplification and sequencing of the 5′-UTR region that contains the CGG repeat. We amplified genomic DNA from both WT males and females, carrier females and affected males. The gel-purified *Fmr1* PCR amplicon was sequenced and no expansions were detected.

### RNA-seq

To identify the potential disease-causing variant, we performed deep RNA-seq on whole cerebella of WT and affected 5-week-old WF males. We chose an age of 5 weeks to ensure that PCs were still present in normal numbers. Based on prior observations (La Regina et al., 1992; Tolbert et al., 1995), PC counts and molecular layer thicknesses of 6-week-old *shaker* rats and age-matched controls were not significantly different. We have previously demonstrated, in a different rodent model of cerebellar ataxia, that the expressions of *Calb1*, *Pcp2* and *Grid2*, as well as that of some other genes highly expressed in PCs, significantly reduce as the disease phenotype progresses (Dansithong et al., 2015; Hansen et al., 2013). Analyses of *Calb1*, *Pcp2* and *Grid2* transcript abundance by quantitative PCR (qPCR) demonstrated that these PC transcripts were not significantly reduced in cerebella of WF *shaker* rats at 5 weeks of age (Fig. S1).

RNA quality was high, with 89 million and 102 million raw reads for WT and affected rats, respectively, and with 96% alignment of sequences. The RNA sequence revealed five variants in our 26-Mbp region of interest from 133 Mbp to qter (the end of the long arm of the chromosome) (Table 1): two intergenic SNPs, two intergenic deletions, and one DNA variant leading to an amino acid (aa) change. The non-synonymous substitution c.33C>T was found in *Atb2b3*, which encodes PMCA3, a plasma membrane Ca^2+^ ATPase transporter that is abundant in rat brain (Ensembl transcript ENSRNOT00000024025, Rat RGSC3.4). The resulting aa change was found at codon 35, substituting an arginine for a cysteine (p.R35C). The variant was well supported by 286 reads with a 99.3% variant allele frequency based on read counts with a *P*-value of 3.1791E^−166^ found in all affected and none of the WT transcripts. RNA expression levels across the candidate gene interval, including those of *Atp2b3*, were not different for WT and mutation carriers at 5 weeks of age (Fig. S2).

**Table 1.**
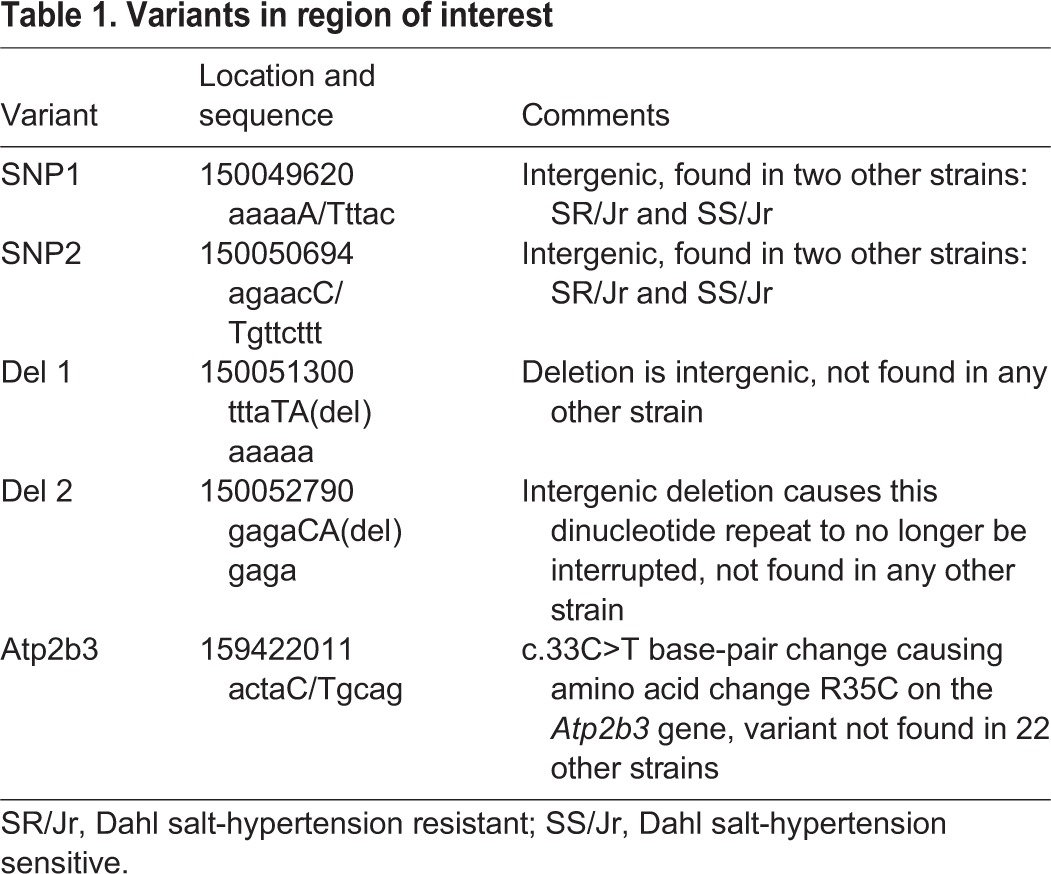
**Variants in region of interest**

**Fig. 2.**
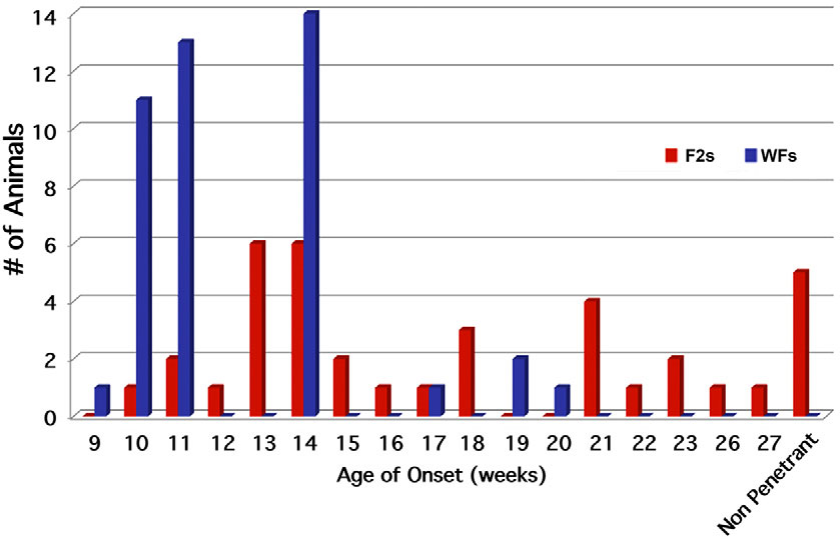
**Age-of-onset graph for Wistar Furth (WF) and F2 male offspring.** Graph represents 105 WF and 92 F2 males. All male progeny were aged to 36 weeks of age before euthanization. 100% of WF and 95% of F2 mutation carriers exhibited an affected phenotype by 36 weeks of age. ‘Non-penetrant’ was defined as mutation-positive but not exhibiting a phenotype by 36 weeks of age. Reduced penetrance was observed only in the F2 cohort.

Once the *Atp2b3* variant was identified, all animals were genotyped. 100% of all mutation-positive rats in the WF background exhibited an affected phenotype by 20 weeks of age. A total of 105 WF and 92 F2 males were produced. All male progeny was aged to 36 weeks of age before euthanization. In the F2 background, 95% of all mutation carriers exhibited an affected phenotype by 36 weeks of age. The phenotype of these affected hybrid progeny was the same as that of affected WF rats. We concluded that the 5% (5/92) of mutation carriers that did not exhibit a phenotype by 36 weeks of age were likely non-penetrant (Fig. 2).

Reverse-transcriptase (RT)-PCR of the entire coding region for *Atp2b3* was performed to rule out other mutations that might affect transcription or exon splicing. No alterations were detected (data not shown).

### Genetic evaluation of *Atp2b3*

We performed genomic PCR of DNAs from six rat strains [Brown Norwegian (BN), SD, F344, Long-Evans, WF and Wistar Kyoto] and found that the arginine encoded by codon 35 was conserved in all. This was also true by *in silico* analysis of the genomes of 16 additional rat strains. In addition, R35 was evolutionarily conserved in all of the 48 sequenced taxa, including humans (Fig. S3).

The human and rat *Atp2b3* genes are 89% identical at the cDNA level and 98% identical at the protein level. Human and rat PMCA3 is almost identical, with the exception of an additional exon in rat composed of 38 aa at the C-terminal end, at c.3343, p.1115 (Ensembl transcript ID ENSRNOT00000078100, A0A0G2K9Q6).

Because protein-variant scoring algorithms are optimized for human proteins, we utilized human PMCA3 for *in silico* analysis of the aa variant. This analysis was performed using a suite of bioinformatics tools: PolyPhen-2, SIFT, PROVEAN, Mutation Taster and CADD. PolyPhen-2 predicted the variant as benign, in direct contrast to SIFT, PROVEAN, Mutation Taster and CADD, which all predicted it as a deleterious or disease-causing mutation. This variant is not known in humans (Table S2).

### *In vitro* characterization of PMCA3

**Fig. 3.**
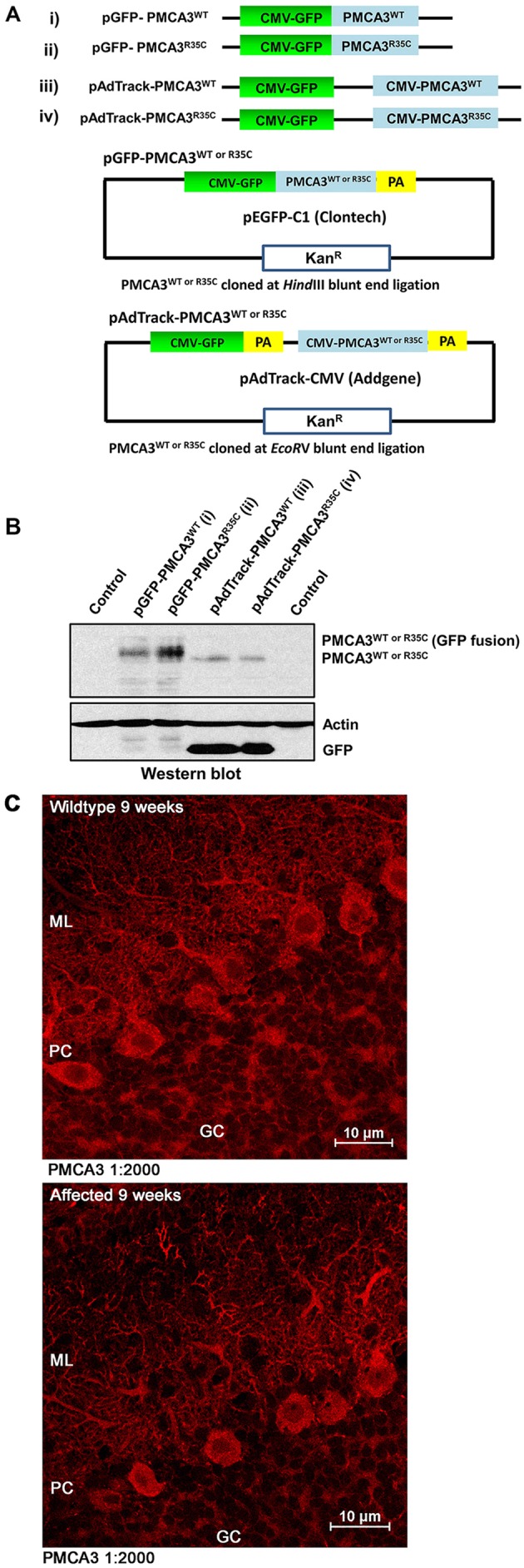
**Characterization of PMCA3.** (A) Schematic representation of GFP-tagged PMCA3 clones. GFP-tagged wild-type (WT) PMCA3 or PMCA3^R35C^ (i, ii) or non-tagged (pAdTrack) WT PMCA3 or PMCA3^R35C^ (iii, iv) with an independent GFP cassette under the transcriptional control of *CMV* promoters are shown. The bottom images show a plasmid map delineating restriction enzymes used to clone the insert, and the selecting antibiotic used. (B) Western blot of SH-SY5Y cell lysates following transfection by GFP-tagged PMCA3 plasmids. Control lanes represent untransfected cell extract. Western blot results confirmed the expression of WT PMCA3 or PMCA3^R35C^ recombinant PMCA3. The experiment was replicated three times. (C) Twelve (10 μm) midline cerebellar sections of each group – WT and *shaker* WF rats – were analyzed at 9 weeks age (*n*=2). Sections were stained with an antibody to rabbit polyclonal anti-PMCA3 ATPase (1:2000) and counterstained with DAPI. PMCA3 staining shows no difference between WT and *shaker* rats. ML, molecular layer; PC, Purkinje cells; GC, granular cell layer.

SH-SY5Y cells were transfected with expression plasmids encoding the WT PMCA3 or PMCA3^R35C^ protein, with an independent *CMV*-*GFP* cassette (Fig. 3A). Construct expression was analyzed 48 h post-transfection by western blot analysis; blots were then sequentially stained with antibodies against PMCA3, GFP and β-actin. GFP and β-actin staining was utilized to verify uniform loading of wells. Western blot results confirmed the expression of WT PMCA3 or PMCA3^R35C^ recombinant PMCA3 at equal levels (Fig. 3B).

### Immunocytochemistry for PMCA3

Immunocytochemical staining of PMCA3 in frozen cerebellar tissue sections revealed no differences between WT and affected male rats at 9 weeks of age (Fig. 3C).

### Ca^2+^ handling by exogenous WT PMCA3 or PMCA3^R35C^

To selectively explore the ability of the mutated PMCA3 pump to handle cellular Ca^2+^ fluxes in living cells, the constructs encoding the splicing variants *a* and *b* of the PMCA3 pump were employed. Both the full-length *b* and the C-terminally truncated isoform *a* of the pump are significantly expressed in cerebellar neurons (Carlson et al., 2009; Eakin et al., 1995). They only differ by the presence of a premature stop codon in the *a* variant, which is caused by a shift in the reading frame during the splicing process that truncates the C-terminal tail of the pump in the middle of the calmodulin-binding domain (Brini and Carafoli, 2009). The loss of about half of the calmodulin-binding domain compromises its ability to auto-inhibit the pump, which becomes considerably more active in the resting state than the original full-length variant. The plasmids encoding isoform *a* of a GFP-tagged (GFP) and a non-tagged (AdTrack) WT PMCA3 or PMCA3^R35C^ were introduced into HeLa cells along with the plasmid encoding the recombinant Ca^2+^-sensitive photoprotein aequorin (cytAEQ). The same procedures were also applied to the *b* variant of the pump and were expressed in HeLa cells in the WT and the mutant form.

**Fig. 4.**
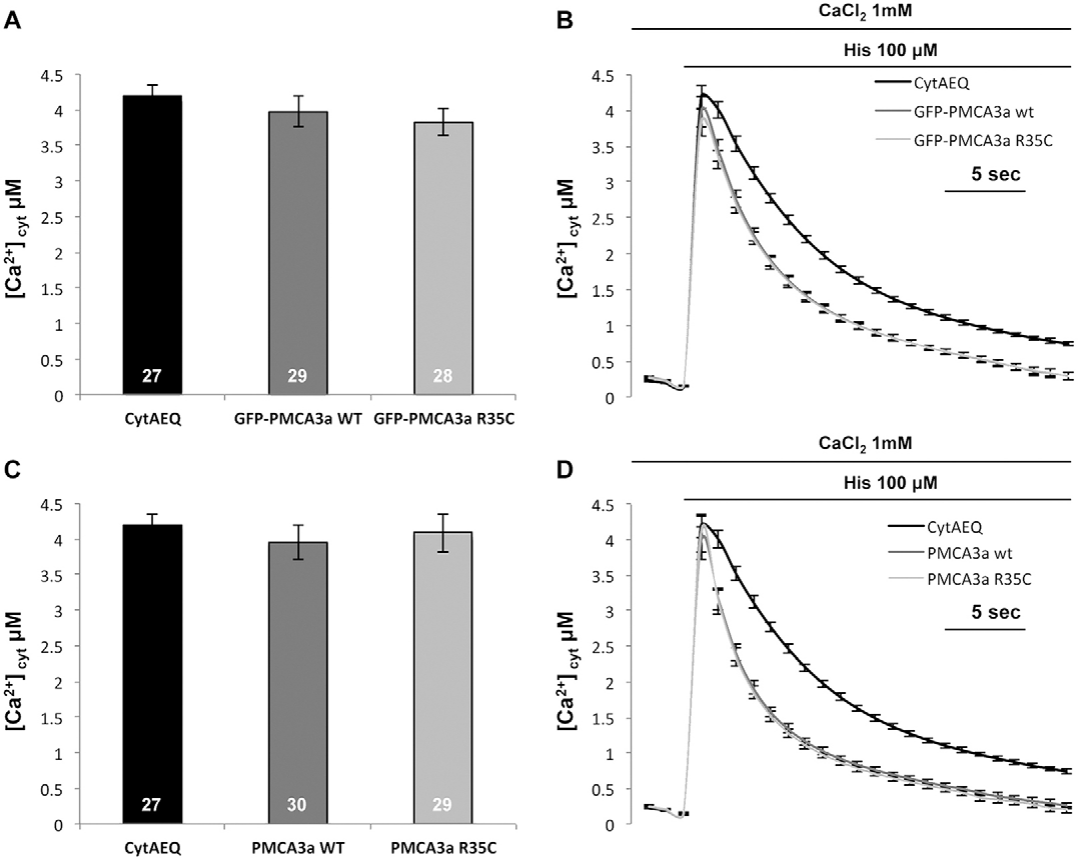
**Cytosolic Ca^2+^ measurements in HeLa cells overexpressing the GFP-tagged and untagged wild-type (WT) and R35C mutant PMCA3a variant.** HeLa cells were co-transfected with cytAEQ and the expression plasmid for the WT and the R35C mutant GFP-tagged and untagged PMCA3a variant. Average peak values (A,C) and cytosolic Ca^2+^ transients (B,D) were recorded following 100 μM histamine (His) stimulation. Bars in panels A and C represent mean [Ca^2+^] values upon stimulation (μM; ±s.e.m.). Peak values in µM are: 4.20±0.15, *n*=27 for cytAEQ, 3.97±0.21, *n*=29 for GFP-tagged WT PMCA3a, and 3.82±0.18, *n*=28 for GFP-tagged R35C PMCA3a; 3.95±0.23, *n*=30 for un-tagged WT PMCA3a, and 4.09±0.26, *n*=29 for un-tagged R35C PMCA3a. The experiment was replicated three times.

**Fig. 5.**
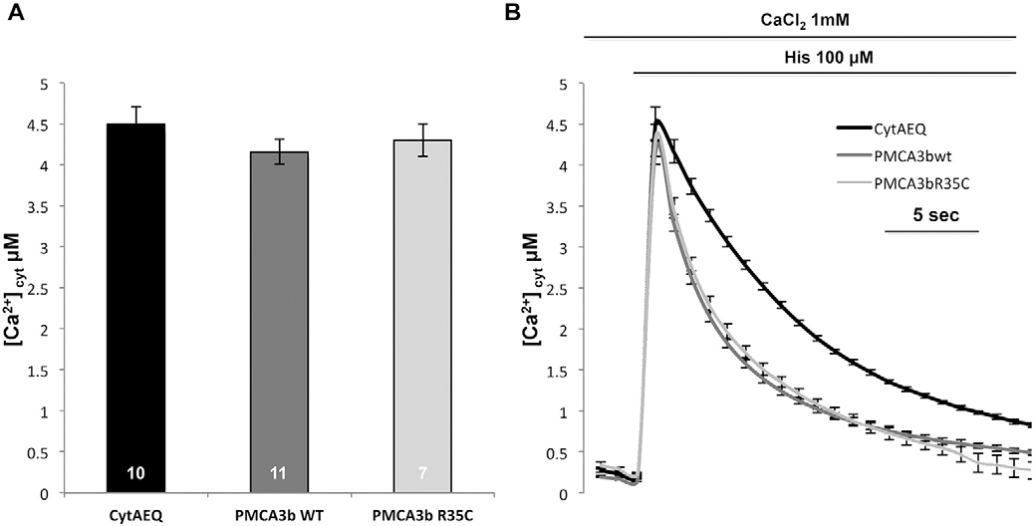
**Cytosolic Ca^2+^ measurements in HeLa cells overexpressing the untagged wild-type (WT) and R35C mutant PMCA3b variant.** HeLa cells were co-transfected with cytAEQ and the expression plasmid for the WT and the R35C mutant untagged PMCA3b variant. Average peak values (A) and cytosolic Ca^2+^ transients (B) were recorded following 100 μM histamine (His) stimulation. Bars in panel A represent mean [Ca^2+^] values upon stimulation (μM; ±s.e.m.). Peak values in µM are: 4.50±0.21, *n*=10 for cytAEQ, 4.16±0.14, *n*=11 for un-tagged WT PMCA3b, and 4.30±0.20, *n*=7 for un-tagged R35C PMCA3b. The experiment was replicated three times.

To study the effect of the R35C mutation on the activity of the overexpressed pump, we monitored cytosolic Ca^2+^ transients generated upon stimulation of HeLa cells with histamine, an inositol 1,4,5-trisphosphate-linked agonist that induces Ca^2+^ release from intracellular stores, and the consequent influx of the Ca^2+^ from the extracellular ambient through channels activated by their emptying (the SOC channels). The statistical analysis and the average traces shown in Fig. 4 indicate that clearance of the Ca^2+^ transient, which was greatly accelerated in both cases with respect to control cells only transfected with cytAEQ, did not differ in cells overexpressing WT or PMCA3^R35C^. Average peak values of the transients were essentially the same in control cells and in cells in which GFP-tagged or un-tagged PMCA3 isoform *a* was overexpressed. Similar results were obtained for the full-length *b* variant of the pump (Fig. 5). Thus, the R35C mutation failed to significantly affect the ability of the pump to counteract cytosolic Ca^2+^ transients.

### Effect of the R35C variant on the basal activity of the PMCA3 pump

To study the effect of the R35C mutation on the basal activity of the PMCA3 pump, a yeast-based functional assay was employed (Ton and Rao, 2004). In this assay, the lethal phenotype of triple-mutant K616 yeast cells in Ca^2+^-free conditions is rescued by the ectopic expression of active Ca^2+^ pumps. The coding sequences of the WT or the mutant full-length PMCA3 pump were inserted in the galactose-inducible plasmid pYES, and transformed in yeast K616 cells.

As a positive control, we used a C-terminally truncated *Atp2b3* mutant (PMCA3-ΔC_ter_), lacking the last 158 C-terminal residues, that is constitutively active because it is not auto-inhibited by the C-terminal tail of the pump. The constitutively active pump variant is a suitable positive control in the assay. Two other mutants were tested. An ATPase-dead mutant, produced by replacing the catalytic aspartate with an alanine (PMCA3 D465A), was used to verify the detrimental effects of a known PMCA-inactivating mutation. A W1104A mutant was generated by site-directed mutagenesis and used as another positive control because it promotes the basal activity of the protein (Penheiter et al., 2002), probably by slowing its inactivation rate.

**Fig. 6.**
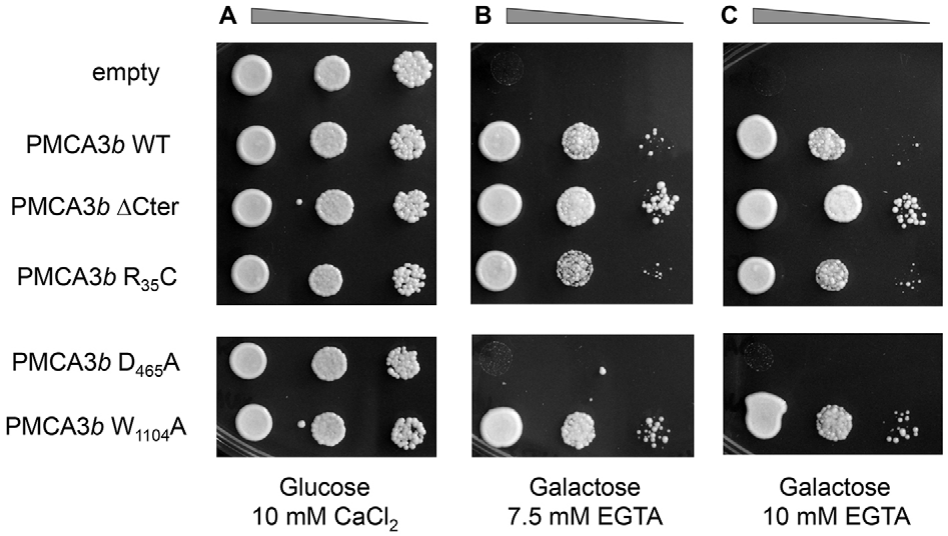
**Functional complementation assay in K616 yeast cells.** Yeast K616 cells were transformed with pYES2-derived vectors carrying one of the following PMCA3b variants: wild type (WT), C-terminally truncated (PMCA3 ΔC_ter_), R35C mutant, D465A mutant and W1104A mutant. Exponentially growing cells have been serially diluted and spotted on glucose+CaCl_2_ medium (A), galactose+7.5 mM EGTA (B) and galactose+10 mM EGTA (C). Cells carrying empty plasmid or expressing constitutively active PMCA3 ΔC_ter_ are negative and positive internal controls of the functional assay, respectively. The experiment was replicated three times.

Our assays included growth controls of yeast transformed with the different *Atp2b3* constructs in a Ca^2+^-containing permissive medium (Fig. 6A) and in an inducible Ca^2+^-free medium (EGTA 7.5 or 10 mM) (Fig. 6B and C, respectively). As expected, the constitutively active PMCA3-ΔC_ter_ mutant rescued the full viability of K616 yeast cells in Ca^2+^-depleted medium (Fig. 6B,C, third row), whereas the auto-inhibited WT *Atp2b3b* (PMCA3b) restored it only partially (Fig. 6B,C, second row). Notably, the recovery of the yeast with the R35C mutation in the absence of Ca^2+^ was not unlike the WT, suggesting that the aa substitution did not affect the ability of the full-length PMCA3 pump to eject Ca^2+^ in the resting, not activated, condition (Fig. 6B,C, compare the second with the fourth rows). As expected, the ATPase-dead D465A control mutant failed to rescue the viability of the K616 yeast cells (Fig. 6B,C, fifth row). Also as expected, the W1104A mutant, which removed the auto-inhibition of the pump, increasing its activity, produced a yeast viability phenotype that was only slightly less pronounced than that observed for the ΔC_ter_ mutant. Thus, the R35C mutant of the PMCA3 was active and properly auto-inhibited in its basal, unstimulated state.

## DISCUSSION

### General

The *shaker* mutation arose spontaneously in an outbred SD strain and was subsequently transferred to an inbred WF background (Clark et al., 2000) owing to fertility problems. It was inferred that the mutation was X-linked based on segregation studies. Wider hind-limb stance for carrier females versus WT rats was attributed to mild loss of PCs, as determined by immunofluorescent staining for calbindin (Clark et al., 2000; Tolbert et al., 1995). This phenotype was originally described as the ‘mild’ trait of the *shaker* rat (La Regina et al., 1992; Tolbert et al., 1995). Utilizing DNA markers on the X chromosome in an F2 intercross, we confirmed X-linked inheritance.

We found evidence for linkage of *shaker* to chromosome Xq36 to qter. The combination of genetic mapping in an F2 cross and analysis of pre-symptomatic cerebellar transcriptomes led to identification of a likely pathogenic DNA variant. Later disease onset in F2s and the rare presence of non-penetrance highlight the effects of genetic background on PC dysfunction and degeneration. The candidate mutation segregated with disease in all affected animals and the aa change was found in an evolutionarily conserved region. The arginine at codon 35 is conserved in 22 different rat strains.

### Phenotype

As the name implies, the *shaker* phenotype is characterized by gait ataxia and total-body tremors. On previous examination by Clark et al., gait evaluation showed abnormalities in gait symmetry, stride length and stride width (Clark et al., 2000). The walking tracks of carrier females were reported as unchanged (Clark et al., 2000).

### Essential tremor

The phenotype of the *shaker* rat was reminiscent of severe essential tremor (ET) rather than cerebellar ataxia in early disease stages, but progressed to significant gait ataxia later. The associations between ET and ataxia of gait and balance are well known. In many patients, tremor is not the only feature of ET, and can be accompanied by neuropsychiatric changes and deficits of gait and balance (Arkadir and Louis, 2013; Louis and Okun, 2011). ET is one of the most common genetic neurological disorders and an inheritance pattern consistent with autosomal-dominant inheritance is seen in many families (Gonzalez-Alegre et al., 2014; Tanner et al., 2001). The main clinical manifestation of ET is postural tremor, but involvement of the extremities, head and trunk might be seen as well. Tremors can be aggravated by emotions, physical exhaustion and hunger, as well as temperature variations. Pathologic reports have identified two distinct types. The first shows cerebellar degeneration with significant PC loss; the second, Lewy bodies confined to the locus ceruleus (Louis, 2009).

### X-chromosomal location and human disease

We mapped the *shaker* locus using an F2 intercross of male WF and BN rats with the assumption that presence of WF alleles would denote proximity to the *shaker* locus. We used 44 DNA markers and mapped *shaker* distal to DXRat21 located at 133 Mbp (rat genome version RGSC3.4). The synteny region between human and rat includes the *Fmr1* gene. Fragile X-associated tremor/ataxia syndrome is a human adult-onset neurodegenerative disorder caused by an expanded trinucleotide repeat in the *FMR1* gene (Hagerman et al., 2001). We excluded a repeat expansion in this candidate gene because an expansion would not have been easily detected by RNA sequencing.

X-linked recessive spinocerebellar ataxias are a heterogeneous group and, to date, five have been reported. SCAX1 (OMIM#302500, http://omim.org/entry/302500) was initially described by Bertini et al. as a neurological disorder characterized by hypotonia at birth, delayed motor development, difficulty standing, cerebellar fits (reminiscent of ET observed in the *shaker* rat), dysarthria, and slow eye movements followed by ataxia in the first years of life (Bertini et al., 2000). It is now known to be caused by a mutation in the *ATP2B3* gene, encoding PMCA3 (Zanni et al., 2012). Precise genetic locations for SCAX2 (Malamud and Cohen, 1958) and SCAX3 (Schmidley et al., 1987) are unknown. SCAX4 (Farlow et al., 1987) was excluded from Xq26-qter by genetic linkage analysis and SCAX5 was excluded from the SCAX1 region in Xq25-q27.1 (Zanni et al., 2008).

### Variant detection by transcriptome analysis

We hypothesized that transcriptome analysis at a stage prior to PC loss would likely identify a loss-of-function allele by deep RNA-seq in cerebellar RNAs, and that the number of variants would be greatly constrained by our refined linkage mapping. Indeed, only few variants were found (Table 1). The most likely variant was a missense change in the *Atp2b3* gene, a gene that is mutated in SCAX1.

The changed aa is located in the N-terminal portion that protrudes into the cytosol. The PMCA3 protein belongs to the family of P-type ion-transport ATPases characterized by the formation of an aspartyl phosphate intermediate during the reaction cycle. Mammalian plasma-membrane calcium ATPases are encoded by at least four separate genes. Expression of these four genes is regulated during development and in a tissue- and cell-type-specific manner.

Several bioinformatic tools (PolyPhen, SIFT, PROVEAN, Mutation Taster and CADD) were used to assess the impact of PMCA3 aa change on protein function. These analyses included change in protein structure and function (PolyPhen, Mutation Taster), conservation of the changed aa residue across species (SIFT, Mutation Taster), and potential effects on mRNA expression or splicing (Mutation Taster) (Schwarz et al., 2010), as well as estimation of the relative pathogenicity of the variant (CADD) (Kircher et al., 2014). PolyPhen predicted this mutation as benign. In contrast, the newer programs SIFT, PROVEAN, Mutation Taster and CADD predicted the variant to be damaging.

Additional evidence came from the fact that we did not detect the variant in an additional 22 rat strains and that this variant was not found in the 60K human exome project (http://exac.broadinstitute.org/gene/ENSG00000067842, accessed January 18, 2016).

Codon 35 was well covered, with a total allele count of >36,000 for neighboring codons.

### Functional characterization

Because the possibility existed that PMCA3^R35C^ was just a rare private mutation that had arisen in the WF strain maintained in Saint Louis, we embarked on a series of *in vitro* and functional experiments to evaluate the biological consequence of the variant. Presence of R35C did not affect *Atp2b3* transcript abundance (Fig. S2) or localization in PCs (Fig. 1B). We then investigated whether it altered functioning of the PMCA3 calcium pump. The disease-linked PMCA3^G1107D^ mutation had shown dramatic alteration of Ca^2+^ transients (Zanni et al., 2012). The pump was studied in model cells by exogenous protein expression of WT PMCA3 and PMCA3^R35C^. The function of PMCA^R35C^ was virtually identical to the WT protein (Fig. 5). An additional yeast complementation assay likewise did not show any difference between the WT and R35C mutant (Fig. 6). Thus, PMCA3^R35C^ was functionally normal, both under conditions in which it was maximally activated and in conditions with a low resting concentration of Ca^2+^ in the cytosol. Therefore, the substitution at codon 35 did not drastically impair the Ca^2+^ extrusion ability of the pump.

Nonetheless, it is still possible that the pump variant identified in *shaker* is causative of the neurological phenotype. In addition to their function in controlling basal Ca^2+^ levels, PMCAs can serve as signaling molecules (Brini, 2009). These functions might include the regulation of the cytoskeleton through interaction with 14-3-3 proteins via the cytosolic N-terminal tail of the pump (Linde et al., 2008), a domain that differs significantly among isoforms (Brini and Carafoli, 2009). PMCAs are also likely involved in local Ca^2+^ signaling via interaction with caveolin-1 (Daniel et al., 2006). Although we did not detect major changes in subcellular localization, it is possible that more subtle changes exist in PCs. A single-residue substitution could affect the transfer of the PMCA pump to the plasma membrane, because a change in one aa in transmembrane domain 5 caused PMCA to remain in the endoplasmic reticulum (Guerini et al., 2000). Second, it is well known that a number of proteins interact with the pump and affect its function (Linde et al., 2008), such as the 14-3-3 (epsilon) protein at the N-terminal region. Most of these proteins interact with the C-terminal tail of the pump (DeMarco and Strehler, 2001), such as CaM (calmodulin), NOS-1 (nitric oxide synthase 1), CASK (Ca^2+^/calmodulin-dependent serine kinase), NHERF2 (Na+/H+ exchanger regulatory factor 2) and several members of the MAGUK (membrane-associated guanylate kinase) family; others interact with the central body of the PMCA pump, such as RASSF1 (a Ras effector protein) (Armesilla et al., 2004).

PMCAs are also part of a postsynaptic complex formed by Homer as well as metabotropic glutamate receptors (mGluRs) and inositol triphosphate receptors (InsP3Rs; ITPRs) (Sgambato-Faure et al., 2006). The levels of mGluR1 and InsP3R1 are reduced in PCs in several rodent models of ataxia. Similarly, late-onset human degenerative ataxias with pure cerebellar phenotypes are associated with disrupted Ca^2+^- and ITPR-dependent pathways (Schorge et al., 2010). The importance of maintaining calcium homeostasis and signaling with precision might explain why a subtle and currently undetectable defect of the PMCA3 pump could generate a severe cerebellar phenotype. Further studies are required to explore the possibility that the N-terminal mutation of the *shaker* rat PMCA3 pump affects its interaction with still-unidentified partners that could have a functional role in PC degeneration.

On the other hand, the differentiation between disease-causing variants and rare benign variants might be complex, as shown by the difficulties identifying the SCA31 mutation. A heterozygous single-nucleotide change (-16C>T) in the *PLEKHG4* (puratrophin-1) gene had been identified within the candidate linkage region and had been proposed as the candidate SCA31 mutation, especially because this variant in the UTR of the *PLEKHG4* gene seemed to show *in vitro* functional effects as well (Ishikawa et al., 2005). Subsequently, Ohata et al. reported one patient with ataxia who did not carry the -16C>T transition in a family showing tight linkage with the UTR variant, suggesting that this variant was only closely linked to the causative repeat mutation (Ohata et al., 2006). Further analysis of the now refined genetic candidate region led to the identification of an expanded pentanucleotide repeat within an intron of the *BEAN* and *TK2* genes as the cause of SCA31 (Sato et al., 2009). In the absence of functional *in vitro* effects of the PMCA3^R35C^ variant, the possibility remains that this genetic change is not causative, but very closely linked to the causative *shaker* mutation.

## MATERIALS AND METHODS

### Animals

All animal work was done under an approved IACUC protocol at the University of Utah. Animals were housed 4-5 animals per cage under a 12:12 h light cycle with food and water provided *ad libitum*. Animals were fed Teklad Global Rodent Diet (Denver, CO).

The *shaker* rat colony used in these studies was obtained from a breeding colony at Saint Louis University. This animal model occurred as a spontaneous mutant in the SD strain and the mutation was later moved into the WF strain via cross-intercross methods, owing to fertility problems in the SD strain. We maintain the *shaker* colony in the WF background.

### Genetic mapping

WF *shaker* rats were crossed with BN rats. BN rats were selected for the hybrid cross because they are genetically most distant from all other strains (Canzian, 1997). F1s were generated by mating of affected WF males with BN WT females and in order to reduce cost and animal numbers. F1s were also produced by mating WF affected females with BN WT males, and carrier WF females with BN WT males. BN WT rats were purchased from Charles River (http://www.criver.com/). F2s were generated by breeding affected F1 males with carrier females. Ninety-two F2 males were retained for phenotype observation and genetic mapping.

### DNA isolation and microsatellite marker genotyping

DNA was extracted using the Puregene Genomic DNA Purification Kit (Gentra Systems; Minneapolis, MN) from whole brain tissue or 3-5 mm of tail tip (soft tissue only). We utilized SNPlotyper on the RGD (http://snplotyper.mcw.edu/analyses) to select markers that would differentiate BN from SD and WF. SNPlotyper is an analysis and visualization tool built on the rat SNP genotype data developed by the STAR consortium. We identified 44 markers that differentiated WF and BN alleles. Genotyping of the F2 progeny was performed and a haplotype table generated (Table S1). The total genetic map length was 137 cM. The average distance between markers was 3.2 cM.

We PCR-amplified tail DNA of all F2s and assigned a calling code for each sample based on standards produced from the donor strains. DNAs from WF and BN WT rats were amplified and sequenced for all markers. The amplicons were then utilized as the standard for each marker as illustrated in Table S1.

### RNA-seq

RNA Illumina HiSeq Sequencing was performed on whole cerebella of WT and affected 5-week-old F2 males. Total RNA was isolated using an miRNeasy mini-kit (Qiagen Inc., Valencia, CA) according to the manufacturer's protocol. RNA quality was obtained using the Bioanalyzer 2100 Pico Chip (Agilent, Santa Clara, CA). Samples with an RNA integrity number (RIN) >8 were used for library preparation using Illumina TruSeq RNA Sample Prep v2 (Illumina, San Diego, CA) with oligo-dT selection.

Three biological replicates from each group were analyzed. Samples of WT and affected cerebella were barcoded and run on a single lane to reduce batch effects. Single-end 50-bp reads were generated on a Hiseq 2000 machine at the University of Utah Microarray and Genomic Analysis Shared Resource using Illumina Version 4 flowcells. Reads were then aligned to the rat RGSC3.4 reference genome using Novoalign. We used the SAMTools mpileup on our alignment files, called variants using VarScan2, focusing on variants found on chrX:112,204,377 to qter. Owing to limited annotation of the rat genome, mouse and human genome databases were used to curate missing areas of coverage.

We utilized VarScan2 (Koboldt et al., 2012) (http://varscan.sourceforge.net/) and DESeq2 (Love et al., 2014) (https://bioconductor.org/packages/release/bioc/html/DESeq2.htm) to examine the level of gene expression and mutation analysis. VarScan2 is a platform-independent mutation caller for targeted, exome and whole-genome resequencing data. In contrast to other software packages that utilize probabilistic frameworks, to detect variants and assess confidence in them, VarScan2 utilizes a robust heuristic/statistic approach to call variants that take into account read depth, base quality, variant allele frequency and statistical significance. DESeq2 is a software package that uses a method of estimate variance-mean dependence in count data from high-throughput sequencing assays and tests for differential expression based on a model using the negative binomial distribution.

### *In silico* structural analysis

*In silico* structural analysis of the variants was done using PolyPhen, SIFT, PROVEAN, Mutation Taster and CADD (http://genetics.bwh.harvard.edu/pph/, http://sift.jcvi.org/, http://provean.jcvi.org/, http://www.mutationtaster.org/, http://cadd.gs.washington.edu/, respectively). For evolutionary conservation, protein sequences were downloaded from NCBI (http://www.ncbi.nlm.nih.gov/protein), and the alignments were calculated using ClustalW (http://www.ebi.ac.uk/clustalw2).

### Exclusion of *Fmr1* repeat expansion

Amplification of the 5′ UTR region containing the CGG repeat of the *Fmr1* gene was performed using HotStar Taq Plus (Qiagen Inc., Valencia, CA), with standard genotyping conditions with the following primers: Fmr1F (5′-TCGGCCTCAGTCAGTCTTGCG-3′) and Fmr1R (5′-CTGTCCGGTAGCCCGTTACCT-3′). PCR conditions were as follows: 95°C 5 min, then 35 cycles of 95°C 1 min, 60°C 30 s, 72°C 1 min, followed by a final extension at 72°C for 5 min.

### RNA expression analyses by quantitative RT-PCR

Rat cerebella were removed and flash frozen in liquid nitrogen. Tissues were kept at −80°C until the time of processing. Total RNA was extracted from rat cerebella using the miRNeasy mini-kit (Qiagen Inc., Valencia, CA) according to the manufacturer's protocol. DNAse-I-treated RNAs were used to synthesize cDNA using the ProtoScript cDNA synthesis kit (New England Biolabs Inc., Ipswich, MA). Quantitative RT-PCR was performed using the QuantStudio 12K Flex Real-Time PCR System (Thermo Fisher Scientific, Grand Island, NY) with the Power SYBR Green PCR master mix (Applied Biosystems Inc., Grand Island, NY). PCR reaction mixtures contained SYBR Green PCR master mix and 2.5 µmol primers, and PCR amplifications were carried out for 45 cycles: denaturation at 95°C for 10 s, annealing at 60°C for 10 s and extension at 72°C for 40 s. The threshold cycle for each sample was chosen from the linear range and converted to a starting quantity by interpolation from a standard curve run on the same plate for each set of primers. All gene expression levels were normalized to actin mRNA levels. Primer pairs designed for qPCR are given as forward and reverse, respectively, for all genes: *Calb1*, *Pcp2*, actin, *Grid2* and *Atp2b3* (Table S3). The sample size was *n*=3 for all assays and only male rats were utilized.

### Genotyping of *Atp2b3* exon 3 on genomic DNA

Amplification of *Atp2b3* exon 3 was performed with standard PCR conditions with the following PCR primers: RS-2A (5′-CCACCCGTCCCTAGCTATT-3′) and RS-2B (5′-TCTGTGTCCTTCATGGGTCA-3′) followed by Sanger sequencing utilizing the same primers. PCR conditions were as follows: 95°C 5 min, then 5 cycles 95°C 1 min, 57°C 30 s, 72°C 1 min, followed by 30 cycles of 95°C 1 min 30 s, 55°C 30 s, 72°C 1 min, and final extension at 72°C for 5 min.

### Genotyping of *Atp2b3* via RT-PCR

Amplification of the *Atp2b3* coding region was performed using cDNA with eight individually paired PCR primers (RT1 through RT8) listed in Table S3. PCR conditions were as follows: 95°C 5 min, then 5 cycles 95°C 1 min, 60°C 30 s, 72°C 1 min 30 s, followed by 30 cycles of 95°C 1 min 30 s, 65°C 30 s, 72°C 1 min 30 s, and final extension at 72°C for 5 min.

### Cell culture and transfection

SH-SY5Y cells were cultured and maintained in DMEM media containing 10% fetal bovine serum (FBS). For the overexpression of proteins, the cells were transiently transfected with DNA constructs using Lipofectamine 2000 reagent (11668-027, Invitrogen, Grand Island, NY) according to the manufacturer's protocol. 48 h post-transfection, the cells were harvested for western blot analyses. We chose to use pAdTrack-*CMV* (Addgene, Cambridge, MA) because it is a vector used for the expression of transgenes when a GFP tracer is desired.

### Preparation of protein lysates and western blot analyses

Cellular extracts were prepared by the single-step lysis method (Paul et al., 2011). SH-SY5Y cells [CRL-2266, American Type Culture Collection (ATCC), Manassas, VA] were harvested and suspended in SDS-PAGE sample buffer (Bio-Rad, Hercules, CA) and then boiled for 5 min. Equal amounts of the extracts were subjected to western blot analysis to determine protein levels using the antibodies listed below. Protein extracts were resolved by SDS-PAGE and transferred to Hybond P membranes (Amersham Bioscience, Piscataway, NJ). After blocking with 5% skim milk in 0.1% Tween 20/phosphate buffered saline (PBS), the membranes were incubated with primary antibodies in 5% skim milk in 0.1% Tween 20/PBS for 2 h at room temperature or overnight at 4°C. After several washes with 0.1% Tween 20/PBS, the membranes were incubated with the corresponding secondary antibodies conjugated with HRP in 5% skim milk in 0.1% Tween 20/PBS for 2 h at room temperature. Following three additional washes with 0.1% Tween 20/PBS, signals were detected by using the Immobilon Western Chemiluminescent HRP Substrate (Millipore, Temecula, CA) according to the manufacturer's protocol. The following antibodies were used throughout the study: rabbit anti-calcium PMCA3 ATPase (ab3530, 1:3000, Abcam Inc., Cambridge, UK), mouse monoclonal anti-calbindin-D-28K (C9848, 1:5000, Sigma Inc., St Louis, MO) and mouse monoclonal anti-GFP (sc-9996, 1:5000, Santa Cruz Inc., Dallas, TX). To control for protein quality and loading, the membranes were reprobed with monoclonal anti β-actin (A3854, 1:10,000, Sigma Inc., St Louis, MO). Secondary antibodies were: donkey anti-rabbit IgG-HRP (sc-2057, 1:5000, Santa Cruz Inc., Dallas, TX) and goat anti-mouse IgG-HRP (A2304, 1:5000, Sigma Inc., St Louis, MO). The cell line was recently authenticated and tested for mycoplasma contamination.

### Immunohistochemistry

Rats were deeply anesthetized with isoflurane, then perfused with ice-cold PBS transcardially. Tissue was quickly removed and submerged into cold 4% paraformaldehyde (PFA) (Electron Microscopy Sciences, Hatfield, PA) and kept at 4°C overnight. The following day, PFA was replaced with 10% sucrose solution in 1× PBS, and kept at 4°C overnight. The following day, 10% sucrose was replaced with 20% sucrose solution at 4°C overnight. The following day, 20% sucrose solution was replaced with 30% sucrose solution, stored at 4°C, and then mounted in Tissue-Tek O.C.T. Compound (Sakura Finetek, Torrence, CA) and stored at −80°C until the time of sectioning.

Tissue sections were cut into slices of 10 μm or 40 μm and placed on Superfrost Plus microscope slides (Thermo Fisher Scientific, Grand Island, NY). The slides were washed in a 1% SDS solution for 5 min, followed by a wash in 1× PBS for 5 min to remove residual SDS. Slides were then washed four times for 15 min each in a washing buffer consisting of 0.01% Triton X-100 and 0.005% Tween 20 in 1× PBS. Blocking solution was applied and incubated overnight at 4°C. Primary antibodies mouse monoclonal anti-calbindin-D-28K (C9848, 1:500, Sigma Inc., St Louis, MO) or rabbit polyclonal anti-PMCA3 ATPase (ab3530, 1:2000, Abcam, Cambridge, UK) were applied and incubated overnight at 4°C. Slides were washed four times for 15 min each in aforementioned washing buffer to remove residual primary antibodies. Secondary antibodies goat-anti-mouse IgG Dylight 550 (84540, 1:500, Thermo Fisher Scientific, Grand Island, NY) or goat-anti-rabbit IgG Dylight 550 (84541, 1:2000, Thermo Fisher Scientific, Grand Island, NY) was applied and allowed to incubate for 2 h at room temperature. Slides were washed four times for 15 min each with wash buffer to remove residual secondary antibodies. Slides were cover-slipped with Fluorogel II containing DAPI (17985-5051, Electron Microscopy Sciences) and allowed to dry for 24 h. Slides were imaged using Nikon EZ-C1 3.80 Confocal software on a Nikon Eclipse Ti microscope.

### Ca^2+^ measurements with recombinant aequorin

Cytosolic Ca^2+^ concentration was monitored by using the recombinant Ca^2+^-sensitive photoprotein aequorin cytAEQ (Ottolini et al., 2014). Measurements were carried out in a Perkin-Elmer (Perkin-Elmer, Waltham, MA) Envision plate reader equipped with a two-injector unit. The day prior to Ca^2+^ measurements, transfected cells were plated onto 96-well plates. Recombinant cytAEQ was reconstituted by incubating HeLa cells (CCL-2, ATCC, Manassas, VA) for 1-3 h with 5 µM coelenterazine in DMEM supplemented with 1% FBS at 37°C in a 5% CO_2_ atmosphere. After reconstitution, cells were placed in 70 µl of Krebs Ringer Buffer (KRB; NaCl 135 mM, KCl 5 mM, KH_2_PO_4_ 400 mM, MgSO_4_ 1 mM, Hepes 20 mM, pH 7.4) supplemented as indicated in the figures, and luminescence from each well was measured for 1 min. Depending on the experiment, 100 µM histamine or 2 mM CaCl_2_ (final concentration) were injected to generate Ca^2+^ transients. The experiments were terminated by lysing the cells with 100 µM digitonin in a hypotonic Ca^2+^-rich solution (10 mM CaCl_2_ in H_2_O) to discharge the remaining aequorin pool. Output data were analyzed and calibrated with a custom-made macro-enabled Excel workbook. Data are reported as means±s.e.m. Statistical differences were evaluated by Student's two-tailed *t-*test for unpaired samples. A *P-*value of ≤0.05 was considered statistically significant. The cell line was recently authenticated and tested for mycoplasma contamination.

### Functional complementation assay in K616 yeast cells

*Saccharomyces cerevisiae* strain K616 (*Mat α; pmr1::HIS3; pmc1::TRP1; cnb1::LEU2; ade2; ura3*) (Cunningham and Fink, 1994) has been used previously to perform Ca^2+^-ATPase functional complementation assays, and was used in the present study (Baekgaard et al., 2006). Briefly, yeast cells were transformed by standard protocols (Gietz and Woods, 2002) with different pYES2-derived vectors, carrying either WT or mutagenized rat *Atp2b3b* cDNAs. The c.33C>T *Atp2b3* point mutation expressing PMCA3^R35C^ was introduced by using the QuikChange II XL site-directed mutagenesis kit (200521-5; Stratagene, Santa Clara, CA) using primers For (5′-CACGCTGGCAGAACTATGCAGCCTCATGG-3′) and Rev (5′-CCATGAGGCTGCATAGTTCTGCCAGCGTG-3′), and verified by sequencing. Positive clones were selected on minimal synthetic defined (SD) medium (6.7% yeast nitrogen base with ammonium sulphate, 2% glucose) lacking uracil and supplemented with 10 mM CaCl_2_. Functional complementation assays (repeated for at least 3-5 independent clones) were performed by growing yeast cells in SD+CaCl_2_ until mid-log phase, when a similar number of yeast cells were serially diluted and spotted on minimal medium containing glucose (pYES2-PMCA3b repression) or galactose (pYES2-PMCA3b induction) as the carbon source. Addition of 10 mM CaCl_2_, or 7.5 and 10 mM EGTA to glucose or galactose media reflects permissive and nonpermissive conditions for yeast K616 viability, respectively.
